# RECRUITING THE MINORITIZED: AFRICAN AMERICAN BEREAVED DEMENTIA CAREGIVERS

**DOI:** 10.1093/geroni/igad104.0032

**Published:** 2023-12-21

**Authors:** Robbin Frazier, Ashley Millenbah, Sheryl Fairbanks, Warren Wolfe, Zachary Baker

**Affiliations:** University of Minnesota, Center for Healthy Aging and Innovation, Minneapolis, Minnesota, United States; University of Minnesota, Minneapolis, Minnesota, United States; University of Minnesota, Minneapolis, Minnesota, United States; Freelance Journalist, Roseville, Minnesota, United States; Arizona State University, Tempe, Arizona, United States

## Abstract

Central to this project was recruiting and learning from African American people, who are underrepresented in Bereaved Dementia Caregiver research. Initially this project was conceptualized with the intent of recruiting a wide range of people of African or Caribbean descent, given the geographic presence of large contingents of African Immigrants in the Twin Cities of Minnesota. In talking to our community partners prior to launch, we elected to restrict participation to those who identified as, or identified as serving, African Americans specifically. Our recruitment strategies were largely informed by deep community engagement built through academic, personal, and non-profit partnerships over several years. We also sought to synergize recruitment efforts for this project with another effort targeting augmentations of dementia friendliness in local African American churches. In discussing recruitment strategies we also sought to hear from those who may not be those traditionally heard from. For instance, to include younger individuals, we recruited contacts through our local Alzheimer’s Association chapter’s Young Champions group. Likewise, because churches are often a major source of African American participant recruitment, we contacted individuals at the Volunteers of America to recruit. Finally, we intentionally broke our recruitment plan into stages (recruiting a handful of individuals at a time) to ensure that participants could nominate those they thought might be most important to hear from within their own communities. This intellectual humility was essential to doing this work properly and maximizing the potential of our results to help African American Bereaved Dementia Caregivers.

